# CoreGenes3.5: a webserver for the determination of core genes from sets of viral and small bacterial genomes

**DOI:** 10.1186/1756-0500-6-140

**Published:** 2013-04-08

**Authors:** Dann Turner, Darren Reynolds, Donald Seto, Padmanabhan Mahadevan

**Affiliations:** 1 Centre for Research in Biosciences, Faculty of Applied Sciences, University of the West of England, Bristol, BS16 1QY, UK; 2 Bioinformatics and Computational Biology Program, School of Systems Biology, George Mason University, Manassas, VA, 20110, USA; 3 Department of Biology, University of Tampa, Tampa, FL, 33606, USA

**Keywords:** Core genes, Bacteriophage, Taxonomy, Viral genomics, Data mining

## Abstract

**Background:**

CoreGenes3.5 is a webserver that determines sets of core genes from viral and small bacterial genomes as an automated batch process. Previous versions of CoreGenes have been used to classify bacteriophage genomes and mine data from pathogen genomes.

**Findings:**

CoreGenes3.5 accepts as input GenBank accession numbers of genomes and performs iterative BLASTP analyses to output a set of core genes. After completion of the program run, the results can be either displayed in a new window for one pair of reference and query genomes or emailed to the user for multiple pairs of small genomes in tabular format.

**Conclusions:**

With the number of genomes sequenced increasing daily and interest in determining phylogenetic relationships, CoreGenes3.5 provides a user-friendly web interface for wet-bench biologists to process multiple small genomes for core gene determinations. CoreGenes3.5 is available at http://binf.gmu.edu:8080/CoreGenes3.5.

## Findings

### Background

Genes that are common between a set of genomes are known as core genes. Core sets of genes have been used to understand better bacterial genome evolution [1], orthology in viral genomes [2], viral evolutionary complexity [3], and to mine pathogen genomes [4]. Core genes have also been used to investigate the origins of photosynthesis [5], as well as to classify and untangle the taxonomy of bacteriophages [6-8]. With such a myriad of uses for core genes and the growing numbers of whole genome sequences, it is important to provide user-friendly and validated software tools for the determination of these genes from sets of genomes. Originally developed in 2002 [9], CoreGenes, a tool for the identification of shared and unique genes among (small) genomes, has been continually updated and refined in response to user demands [10]. These changes include increased robustness of the tool, as well as the ability to upload custom and proprietary data not deposited in GenBank. The major update to this version is the ability and versatility to batch process multiple pairs of small genomes, freeing the user from repetitive and time-consuming manual entry of genome sets. This is of benefit to users who have several large sets of genomes that they wish to analyze, for example a family of bacteriophages.

Other software tools have been developed for the determination of core genes including mGenomeSubtractor [11], CEGMA [12], nWayComp [13], and GenomeBlast [14]. mGenomeSubtractor and GenomeBlast both use BLAST-based algorithms to identify core genes. Of these, mGenomeSubtractor is primarily intended for use with bacterial genomes, whilst CEGMA is intended primarily for eukaryotic genomes; nWayComp and GenomeBlast are no longer accessible online, as is another genome comparison tool called GOAT [15]. In contrast, CoreGenes has been continuously available online since 2002, and shown to be invaluable in characterizing and re-determining the taxonomy and relationships of bacteriophages based on coding sequences [6,7,16-19]. It is anticipated that this timely update of CoreGenes will enable the analysis of shared proteins among viral and small bacterial genomes in a faster and more efficient manner.

## Implementation

CoreGenes3.5 is implemented using Java, Javascript, and HTML. It uses an iterative BLASTP algorithm that processes a reference genome and multiple query genomes. This is based on the GeneOrder algorithm described previously [20,21]. Briefly, these genomes are retrieved directly from GenBank, or custom-entered by the user, and the gene translations are parsed from the files. Each protein from the first query genome is analyzed against the reference genome proteins using BLASTP, creating a new reference genome which is a subset of the original reference genome and which contains those proteins that meet or exceed the user-defined similarity threshold (BLASTP score). The second query genome is then BLASTP-analyzed against this new reference genome, creating another new reference genome. This iterative process continues until no further query genomes remain. The final output consists of proteins that are common to all the input genomes.

Batch processing of genomes in CoreGenes3.5 is implemented using Javascript and provides the facility to perform comparisons between many pairs of reference and query genomes. Given two lists of genome accession numbers, the script iteratively submits genomes in pairs to the main CoreGenes program such that each reference genome is individually compared with each query genome. When two identical input lists of genome accession numbers are provided, the script results in an ‘all versus all’ comparison, where all genomes are individually compared against each other as both reference and query. Additionally, each genome is compared against itself, providing an internal control where the number of paired proteins should equal the number of protein encoding genes denoted within the genome annotation.

As the BLASTP comparisons are performed *ab-initio* and not pre-computed, CoreGenes3.5 is limited to genome sizes of 2 Mb or less. While CoreGenes3.5 can take larger genomes as input, the time taken to process them also increases. Therefore, it is recommended that users submit genomes with the aforementioned limit.

## Results and discussion

**Figure 1:**
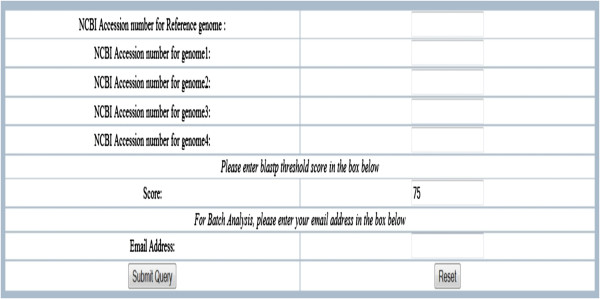
**Web interface for CoreGenes3.5.** A reference genome accession number is entered into the first text field and query genome accession numbers are entered into the subsequent fields. Options for modifying the BLASTP threshold score and an email address are provided.

**Figure 2:**
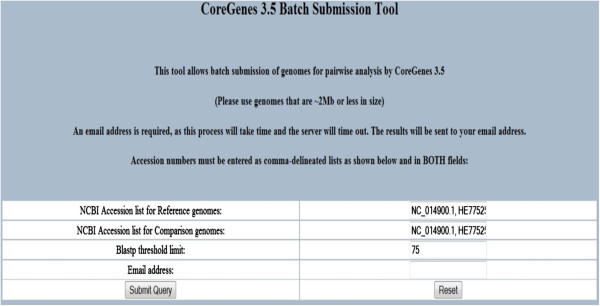
**Web interface for batch processing in CoreGenes3.5.** Reference and query accession numbers are entered in ‘comma delimited’ format. An email address is required so that output tables are emailed to the user following completion of the analysis.

The input to CoreGenes3.5 consists of a reference genome and a query genome. These are entered into the text fields on the web interface as GenBank accession numbers (Figure 1). As noted, the BLASTP threshold score can be changed from its default of “75”. An email address must be entered to which results are sent. For genomes that are not available in GenBank, a link is provided to a custom genome entry page where the user can upload proprietary and unpublished data (these data and results are scrubbed from the server nightly to provide a level of confidentiality). In the batch entry mode (Figure 2), the input consists of two “comma-delineated” lists of GenBank accession numbers in addition to the threshold field. Additionally, CoreGenes3.5 works best with the Mozilla Firefox web browser.

**Figure 3:**
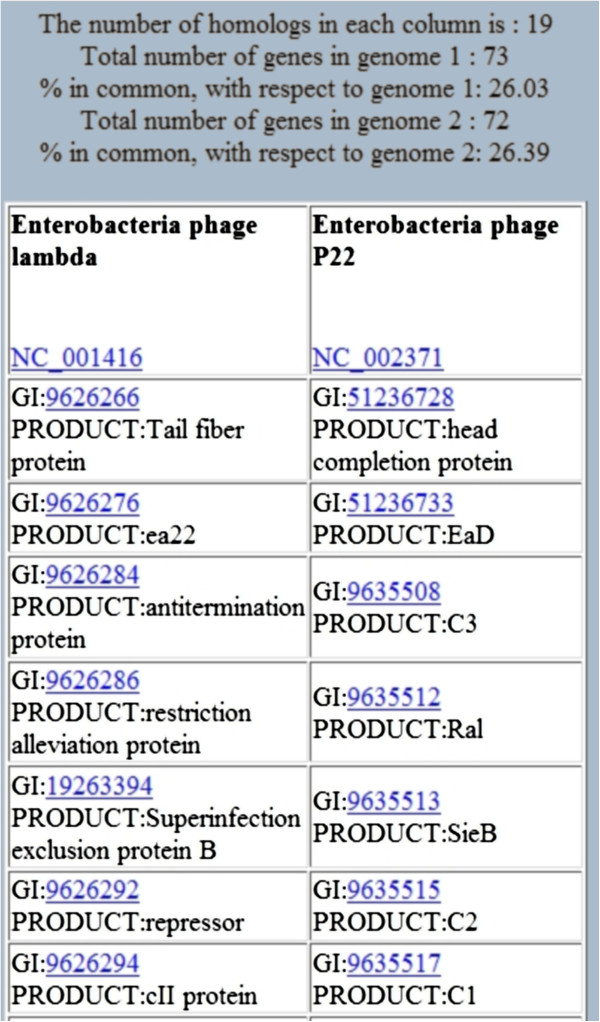
**Partial output of the analysis between the Enterobacteria phages lambda and P22, generated using the batch mode input.** Links (blue) are provided, pointing to whole genome data and specific proteins archived in GenBank. Statistics of how many homologs are found by CoreGenes are summarized at the top of the table.

The output of CoreGenes3.5 is a list of core genes in tabular format (Figure 3). Each gene is linked to its corresponding entry in GenBank. This allows users to identify particular genes of interest for further investigation, provide valuable insights for annotation of function and inform the design of wet-bench studies. In batch processing mode, results tables are emailed to the user in HTML format which can be viewed using any web browser.

The advent and continued development of next generation technologies has substantially increased the throughput and fidelity of genome sequence data. With reducing costs, the number of viral and bacterial genomes deposited in the International Nucleotide Sequence Databases/GenBank has grown rapidly (and continues to do so). It is therefore crucial to continue the development and improvement of novel and existing software tools that can efficiently mine this expanding wealth of sequence data and facilitate comparisons of multiple closely or distantly related genomes.

CoreGenes3.5 is the latest and most versatile update to a user-friendly tool for locating and identifying core genes from viral and small bacterial genomes. Like previous versions of CoreGenes, this newest version will be continually updated in response to demands from the user community. The ability of CoreGenes to deal with larger bacterial genomes is actively being addressed.

## Conclusions

The batch processing feature of CoreGenes3.5 enables researchers to analyze multiple small genomes expeditiously using a web interface. This allows users to data mine the increasing numbers of genomes in sequence databases and to determine quickly the phylogenetic relationships amongst them.

## Availability and requirements

**Project name:** CoreGenes3.5

**Project home page:**http://binf.gmu.edu:8080/CoreGenes3.5

**Operating system(s):** Platform independent

**Programming language:** Java

**Any restrictions to use by non-academics:** License required for commercial usage

## Competing interests

The authors declare that they have no competing interests.

## Authors’ contributions

DT and PM implemented the batch processing in CoreGenes3.5. DT, DR, DS, and PM wrote the manuscript. All authors read and approved the final manuscript.
